# Genicular Artery Embolization for Knee Osteoarthritis: An Interventional Radiology Perspective on Pathophysiology, Imaging Biomarkers and Procedural Strategy

**DOI:** 10.3390/diagnostics16091325

**Published:** 2026-04-28

**Authors:** Alberto Rebonato, Mattia Ruschioni, Luigi Mancini, Luca Mulazzani, Eva Fraternali, Marco Baldini, Nicolò Baiocchi, Daniele Maiettini, Corrado Tagliati, Luca Memè

**Affiliations:** 1 Radiology Department, San Salvatore Hospital, 61121 Pesaro, Italy; mattia.ruschioni@sanita.marche.it (M.R.); luigi.mancini@sanita.marche.it (L.M.); luca.mulazzani@sanita.marche.it (L.M.); 2 Orthopedic Department, San Salvatore Hospital, 61121 Pesaro, Italy; eva.fraternali@sanita.marche.it (E.F.); marco.baldini@sanita.marche.it (M.B.); luca.meme@sanita.marche.it (L.M.); 3 Department of Translation Medicine and for Romagna, University of Ferrara, 44121 Ferrara, Italy; nicolo.baiocchi94@gmail.com; 4 Division of Interventional Radiology, European Institute of Oncology, 20141 Milan, Italy; danielemaiettini@gmail.com; 5 Ospedale di Comunità Maria Montessori di Chiaravalle, AST Ancona, 60033 Chiaravalle, Italy; corrado.tagliati@gmail.com

**Keywords:** genicular artery embolization, knee osteoarthritis, interventional radiology, minimally invasive treatment, angiogenesis, synovial hypervascularity

## Abstract

Genicular artery embolization (GAE) has emerged as a minimally invasive interventional radiology technique for the management of symptomatic knee osteoarthritis (OA), a highly prevalent condition associated with substantial functional impairment and socioeconomic burden. The rationale of GAE is based on superselective embolization of pathological periarticular neovascularization, aiming to modulate synovial inflammation, angiogenesis, and nociceptive signaling while preserving physiological joint perfusion. This narrative review provides an interventional radiology–oriented framework integrating pathophysiological mechanisms, imaging-based patient selection, and procedural strategy. Particular emphasis is placed on the vascular–inflammatory phenotype of OA, MRI-derived biomarkers of synovitis and hypervascularity, and technical aspects of embolization, including embolic agent selection and angiographic endpoints. A structured literature search was performed to identify relevant studies, including prospective trials and randomized controlled studies. Available evidence is critically discussed, with attention to clinical outcomes, safety profile, and current limitations. In addition, practical technical considerations and procedural pitfalls are summarized to provide a clinically applicable perspective. GAE represents a promising therapeutic option for selected patients with knee OA refractory to conservative management. However, further high-quality studies are required to define long-term durability, optimal patient selection, and standardized procedural strategies.

## 1. Introduction

Knee osteoarthritis (OA) is a leading contributor to chronic musculoskeletal pain and long-term functional decline, representing a major challenge for aging societies worldwide. The prevalence of symptomatic knee OA has risen steadily over recent decades, reflecting demographic shifts, increased longevity, and the expanding burden of obesity and metabolic disease [1]. As a result, knee OA has become a principal driver of mobility limitation and loss of independence among older adults.

The clinical expression of knee OA extends well beyond structural cartilage degeneration. While mechanical overload remains an important initiating factor, growing evidence indicates that metabolic dysregulation, low-grade inflammation, and vascular alterations play a central role in disease progression and pain generation. Excess adiposity amplifies joint stress and simultaneously promotes a proinflammatory intra-articular environment through adipokine and cytokine signaling, accelerating synovial activation and subchondral bone remodeling [2].

The cumulative impact of knee osteoarthritis is substantial. In addition to persistent pain and progressive functional impairment, affected individuals often experience reduced participation in daily activities, diminished quality of life, and increased reliance on healthcare services. From a societal perspective, these factors translate into a significant economic burden, with direct and indirect costs exceeding USD 27 billion annually in the United States alone [3].

Management of knee OA is traditionally structured along a stepwise continuum. Conservative strategies—including exercise-based rehabilitation, weight reduction, and pharmacologic analgesia—remain first-line interventions but frequently provide incomplete or transient symptom relief. Although total knee arthroplasty (TKA) offers definitive treatment for advanced disease, a considerable proportion of patients are either unsuitable surgical candidates or elect to defer operative intervention because of medical comorbidities, advanced age, or concerns regarding invasiveness and recovery [4,5]. This therapeutic gap has driven increasing interest in alternative, less invasive treatment approaches.

Parallel to these clinical challenges, the conceptual framework of osteoarthritis has evolved. OA is now increasingly regarded as a disorder of disrupted joint homeostasis, in which mechanical stress, synovial inflammation, angiogenesis, and neurovascular remodeling interact dynamically to sustain pain and functional decline [6]. Within this model, pathological periarticular neovascularization and associated sensory nerve ingrowth have emerged as relevant therapeutic targets.

Genicular artery embolization (GAE) has recently gained attention as a minimally invasive endovascular technique designed to address these vascular and inflammatory mechanisms. By selectively reducing abnormal periarticular hypervascularity, GAE aims to modulate synovial inflammation, suppress angiogenesis-related nociceptive pathways, and alleviate pain while preserving physiological joint perfusion. Early clinical studies and randomized trials suggest that this approach may offer meaningful symptom improvement with a favorable safety profile in carefully selected patients with refractory knee OA [7].

This narrative review explores GAE from an interventional radiology perspective. Emphasis is placed on the biological rationale underpinning the technique, its clinical evolution, relevant vascular anatomy, technical considerations, patient selection, and outcome assessment.

Unlike previous reviews primarily focused on clinical outcomes or evidence synthesis, the present manuscript aims to provide an interventional radiology–oriented framework integrating pathophysiological mechanisms, imaging biomarkers, and procedural strategy. This approach is intended to bridge the gap between biological rationale, imaging-based patient selection, and technical execution in genicular artery embolization.

## 2. Methods—Literature Search Strategy

A structured literature search was performed to identify relevant studies on GAE for knee osteoarthritis. The search was conducted using PubMed/MEDLINE, Scopus, and Web of Science databases, including articles published up to January 2026.

The following keywords and combinations were used: “genicular artery embolization”, “knee osteoarthritis”, “synovitis”, “hypervascularity”, and “interventional radiology”.

Eligible studies included clinical trials, observational studies, systematic reviews, and meta-analyses focusing on the pathophysiology, imaging assessment, technical aspects, and clinical outcomes of GAE. Reference lists of selected articles were also screened to identify additional relevant publications.

Given the narrative nature of this review, no formal systematic selection or quality scoring process was applied; however, emphasis was placed on recent, high-quality, and clinically relevant studies.

## 3. Clinical Origins and Progressive Expansion of GAE

GAE was initially developed in a clinical context unrelated to degenerative joint disease, namely the management of spontaneous recurrent hemarthrosis following total knee arthroplasty. In this setting, selective embolization of hypertrophied synovial vessels proved effective in achieving hemostasis and reducing bleeding recurrence, while preserving periarticular perfusion and without adverse effects on peri-prosthetic tissue viability or prosthetic function [8,9]. These early experiences demonstrated the technical feasibility and safety of superselective embolization within the complex periarticular vascular network of the knee and established the foundational principles later applied to osteoarthritis.

The translational extension of this endovascular approach to knee osteoarthritis (OA) was driven by increasing recognition of synovial hypervascularity and angiogenesis as active contributors to OA-related pain rather than passive bystanders of structural degeneration. This conceptual shift was first translated into clinical practice by Okuno and colleagues, who reported the use of transcatheter arterial embolization in patients with symptomatic mild-to-moderate knee OA refractory to conservative therapy [10]. By targeting angiographically evident periarticular hypervascularity, this proof-of-concept application aimed to modulate the vascular–inflammatory component of OA pain through superselective embolization of pathological neovessels.

Following these seminal observations, GAE rapidly evolved from an exploratory intervention into a structured investigational therapy. Early feasibility studies and small prospective cohorts established procedural reproducibility and initial signals of clinical benefit, thereby providing the rationale for subsequent prospective trials and randomized controlled investigations [10,11]. This progressive transition from translational proof-of-concept to higher-level clinical evidence has contributed to refinement of embolization techniques, embolic material selection, and patient selection strategies.

At present, the evidence base for genicular artery embolization continues to expand, with numerous prospective studies and registered clinical trials exploring refinements in procedural technique, embolic material selection, and application across broader osteoarthritis phenotypes [12,13,14,15,16,17,18,19,20,21,22,23,24].

The expanding body of prospective studies, randomized controlled trials, and longer-term observational data that currently underpins the clinical adoption of GAE—and defines its comparative effectiveness, safety profile, and positioning within contemporary osteoarthritis treatment algorithms—is reviewed in detail in Section 10.

**Figure 1:**
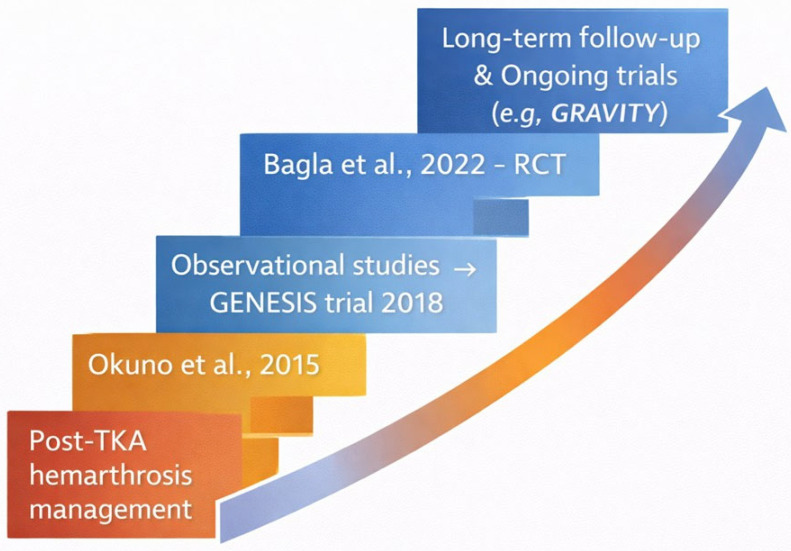
Progressive clinical development of GAE, from its initial use in the treatment of recurrent hemarthrosis after total knee arthroplasty to its application in knee osteoarthritis.

An overview of the clinical origins and progressive evolution of GAE is summarized in Figure 1.

## 4. Pathophysiological Rationale: The Vascular–Inflammatory Pain Phenotype in Knee OA

**Figure 2:**
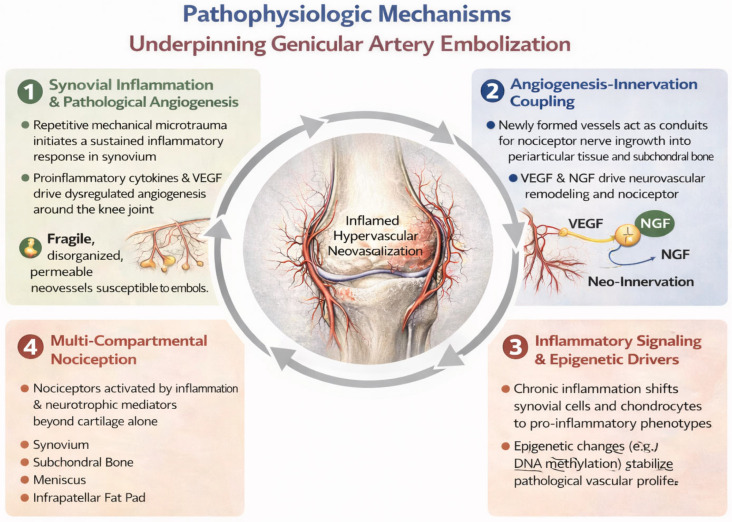
The self-perpetuating cycle linking synovial inflammation, pro-angiogenic mediators (including VEGF and other cytokines), and pathological angiogenesis in knee OA.

Knee OA is increasingly recognized as a whole-joint disorder in which pain and functional limitation are sustained not only by progressive cartilage degeneration but also by synovial inflammation, angiogenic remodeling, and neurovascular sensitization [25,26,27,28]. Mechanical overload and microstructural injury activate synovial and osteochondral stress responses, with consequent macrophage infiltration, synoviocyte proliferation, and release of pro-inflammatory mediators. In a relevant subset of patients, this inflammatory milieu promotes pathological vascular remodeling and amplifies nociceptive signaling, generating a vascular–inflammatory pain phenotype that is not reliably captured by radiographic staging alone [25,26,27,28] (Figure 2).

### 4.1. Synovial Inflammation and Pathological Angiogenesis

Synovitis represents a key symptomatic driver in OA and is associated with increased local production of cytokines and pro-angiogenic mediators, including vascular endothelial growth factor (VEGF) [25,26,27,28,29,30,31,32,33,34,35,36,37]. The downstream effect is the expansion of an abnormal periarticular microvascular network characterized by increased permeability and disorganized microcirculation, which contributes to inflammatory persistence and pain generation [25,26,27,28,29,30,31,32,33,34,35,36,37]. Importantly, these vascular changes are not merely epiphenomena of degeneration but correlate with symptomatic burden and inflammatory OA phenotypes in imaging and histopathologic studies [25,26,27,28,29,30,31,32,33,34,35,36,37].

### 4.2. Angiogenesis–Innervation Coupling and Peripheral Sensitization

Pathological angiogenesis in OA is tightly coupled with sensory nerve sprouting. Newly formed microvessels may act as conduits for nociceptive fibers to extend into tissues that are normally sparsely innervated (synovium, osteochondral junctions, and periarticular soft tissues) [26,27,28]. Proangiogenic and neurotrophic mediators—particularly VEGF and nerve growth factor (NGF)—in synergy with inflammatory cytokines, lower nociceptor activation thresholds and facilitate sustained peripheral sensitization, thereby amplifying pain perception [26,27,28].

### 4.3. Mechanisms Underpinning GAE

From an interventional radiology perspective, GAE is designed to modulate the vascular–inflammatory pain phenotype described above by selectively targeting angiographically identifiable pathological periarticular hypervascularity, rather than structural degeneration such as cartilage loss or osteophyte formation [10,11,25,26,27,28]. The mechanistic rationale is therefore inherently “target-based”: abnormal periarticular blush is treated with superselective embolization to achieve microvascular pruning while preserving physiological perfusion and collateral pathways. These microvessels are characterized by fragile architecture and increased permeability, features that render them amenable to distal embolic pruning when a strict superselective approach is adopted [10,11]. By reducing perfusion to pathological neovascular networks, GAE may attenuate synovial inflammatory activity by decreasing inflammatory mediator delivery and limiting immune-cell trafficking within hypervascular synovial tissue [25,26,27].

## 5. Patient Selection and Pre-Procedural Evaluation

Patient selection for GAE is intrinsically linked to pre-procedural evaluation, including clinical assessment, imaging findings, and exclusion of contraindications. These elements are considered together within a unified framework to ensure appropriate patient selection and procedural planning.

### 5.1. Indications

The clinical rationale for GAE arises from an unmet therapeutic need in patients with knee osteoarthritis who remain symptomatic despite appropriate non-operative management. In routine practice, this population typically includes individuals who have undergone structured lifestyle modification, targeted physical rehabilitation, and pharmacological therapy, yet continue to experience clinically relevant pain and functional limitation. In this context, GAE has been explored primarily in patients for whom surgical intervention is contraindicated, undesirable, or intentionally deferred [7,10]. Contemporary OA management is grounded in a stepwise escalation model, in which treatment intensity is progressively adapted to symptom severity and functional impairment. Minimally invasive procedures are generally considered only after failure of first-line conservative strategies. Within this framework, intra-articular injections are widely used; however, their effects are often transient and variably effective, particularly in patients with active synovial inflammation or complex pain phenotypes. These limitations have prompted growing interest in alternative interventions that target biological mechanisms beyond nociceptive suppression alone, including vascular- and inflammation-directed approaches such as GAE [7,29].

To date, reliable baseline predictors of clinical response to GAE have not been definitively established. Early investigations were frequently limited by small sample sizes, selective enrollment, and insufficient statistical power to support robust subgroup analyses, resulting in heterogeneous and sometimes conflicting findings regarding the influence of demographic and clinical variables on treatment outcomes [10,30,31]. Despite these methodological constraints, multiple studies have consistently reported meaningful pain reduction and functional improvement across a broad spectrum of osteoarthritis severity, encompassing both early-stage and advanced disease defined by radiographic and clinical criteria [32,33].

Emerging evidence suggests that disease stage and symptom profile may influence the magnitude and durability of response. Patients with mild-to-moderate osteoarthritis appear more likely to achieve sustained clinical benefit than those with end-stage disease [34,35]. In addition, higher baseline pain intensity and elevated body mass index have been associated with greater symptomatic improvement in selected cohorts, potentially reflecting an osteoarthritis phenotype characterized by heightened inflammatory and angiogenic activity that may be particularly responsive to vascular-targeted intervention [35,36]. Psychosocial factors may further modulate perceived outcomes; notably, increased baseline pain catastrophizing has been linked to greater reported pain improvement following embolization, underscoring the multifactorial nature of pain response in knee osteoarthritis [30].

Although patient selection criteria continue to evolve, most prospective studies have enrolled individuals older than 40 years with radiographically confirmed knee osteoarthritis (Kellgren–Lawrence grades I–IV), persistent moderate-to-severe pain (VAS ≥ 40 mm) despite at least 3–6 months of optimized conservative therapy, and either contraindications to surgery or a preference to defer operative management [10,11,24,37,38]. Although KL grade 1 may be included, most available evidence primarily focuses on patients with KL grade ≥ 2.

Clinical outcomes following GAE appear to be more favorable in patients with moderate pre-treatment functional impairment and radiographic disease severity. Consistent with the observations reported by Lee et al., who demonstrated higher clinical success rates and greater pain reduction in patients with mild-to-moderate knee osteoarthritis (Kellgren–Lawrence grade II–III) compared with severe disease, we similarly consider that patients with moderate osteoarthritis are more likely to benefit from GAE. This differential response likely reflects the predominant role of synovial inflammation, pathological neoangiogenesis, and associated neoinnervation in earlier disease stages, which are effectively targeted by embolization, whereas pain in advanced osteoarthritis is more strongly driven by irreversible structural damage and subchondral bone pathology, limiting the therapeutic impact of GAE [39].

Collectively, these inclusion parameters support the role of GAE as a therapeutic option for symptomatic patients across a wide range of osteoarthritis severity who have exhausted non-operative treatments, with the potential to delay or avoid arthroplasty.

From a clinical governance perspective, evaluation of candidates for GAE is ideally performed within a multidisciplinary framework, integrating orthopedic, rheumatologic, and interventional radiology expertise. Such an approach facilitates individualized decision-making that balances symptom burden, functional goals, patient expectations, and overall quality-of-life considerations.

### 5.2. Contraindications and Risk Mitigation

Appropriate patient selection remains central to preserving the favorable safety profile of GAE. Although GAE is generally regarded as a low-risk, minimally invasive procedure, specific clinical scenarios warrant exclusion or heightened procedural caution.

Advanced peripheral arterial disease represents a major contraindication, as genicular arteries frequently function as critical collateral pathways in patients with lower-extremity arterial occlusive disease. In this setting, embolization may compromise distal perfusion and exacerbate ischemia [29,37,38].

Active or suspected infection of the knee joint constitutes a contraindication to embolization, as disruption of local vascular supply may interfere with physiological inflammatory and reparative mechanisms, potentially worsening infection or delaying resolution. Comprehensive clinical, laboratory, and imaging evaluation is mandatory to exclude infection prior to treatment, and embolization should be deferred until complete clinical resolution is achieved [14,17,22].

Severe renal dysfunction should be considered a relative contraindication, particularly in patients with advanced chronic kidney disease, given the use of iodinated contrast medium, although the total contrast volume is typically limited [29].

Finally, patients presenting with knee pain in the absence of radiographic or MRI evidence of osteoarthritis are unlikely to benefit from GAE, as symptoms may be driven by centralized pain mechanisms or non-degenerative etiologies. Similarly, fibromyalgia and systemic autoimmune or inflammatory disorders are commonly associated with pain phenotypes that respond poorly to vascular-targeted interventions. In these scenarios, multidisciplinary assessment and detailed patient counseling are essential to avoid inappropriate treatment selection and unrealistic expectations [29]. Detailed aspects of pre-procedural clinical evaluation and patient preparation are further described in Section 7.

A structured summary of principal contraindications, associated risks, and recommended mitigation strategies is provided in Table 1.

## 6. Assessment of Osteoarthritis Severity and Clinical Outcome Evaluation in GAE

Accurate stratification of osteoarthritis severity and systematic assessment of clinical outcomes are central to both patient selection and efficacy evaluation in GAE. Unlike structural interventions aimed at correcting mechanical joint failure, GAE is primarily intended to modulate pain-related inflammatory and vascular mechanisms; therefore, outcome assessment must prioritize clinically meaningful changes in pain, function, and quality of life rather than structural modification alone.

### 6.1. Imaging-Based Severity Classification: Contextual Role in GAE

Conventional weight-bearing radiography remains the most widely available modality for baseline structural assessment of knee osteoarthritis. The Kellgren–Lawrence (KL) grading system continues to be routinely applied in GAE studies due to its historical validation and ease of use across clinical settings [10,11,37,40]. KL grading provides a standardized framework for categorizing disease severity based on joint space narrowing, osteophyte formation, subchondral sclerosis, and osseous deformity.

**Figure 3:**
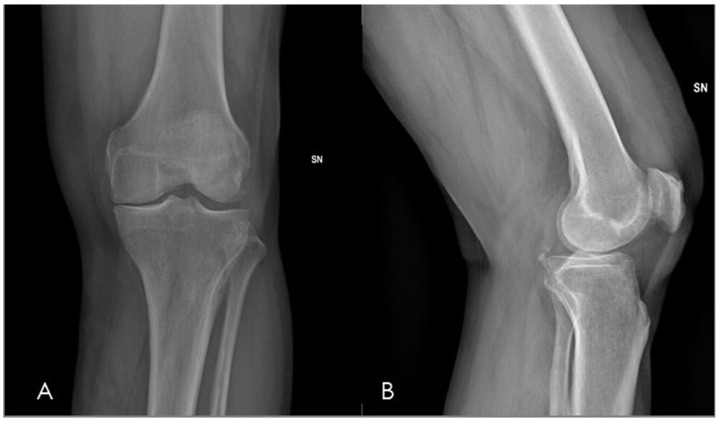
Plain radiographs of the left knee in a 70-year-old male with chronic pain refractory to conservative therapy, including anteroposterior (**A**) and lateral (**B**) views, obtained prior to GAE. The radiograph demonstrates tricompartmental osteoarthritis, characterized by joint space narrowing with associated subchondral cortical sclerosis and early marginal osteophyte formation, corresponding to Kellgren–Lawrence grade III.

However, the clinical relevance of radiographic severity in predicting pain intensity and therapeutic response is limited. Large epidemiological studies have consistently demonstrated a weak correlation between KL grade and symptom burden, with up to half of individuals with radiographic osteoarthritis reporting minimal or no pain [31]. This structural–symptomatic discordance is particularly relevant for GAE, as the procedure does not target cartilage loss or bony deformity but rather pathological synovial hypervascularity and inflammation. For this reason, radiographic grading in GAE should be interpreted primarily as a descriptive staging tool, useful for cohort characterization and comparison across studies, rather than as a determinant of expected clinical response (Figure 3).

### 6.2. MRI as a Biomarker-Oriented Assessment Tool

Several semiquantitative scoring systems have been developed to standardize MRI assessment in osteoarthritis, including WORMS, BLOKS, KOSS, and MOAKS. Among these, the Whole-Organ Magnetic Resonance Imaging Score (WORMS) remains the most frequently employed in OA and GAE-related studies due to its broad anatomical coverage and reproducibility [29,41].

Beyond its diagnostic role, MRI is increasingly recognized as a potential stratification tool for identifying osteoarthritis phenotypes that may preferentially respond to GAE. In particular, imaging features reflecting active synovitis, bone marrow lesions, and increased periarticular perfusion have emerged as candidate biomarkers of a vascular–inflammatory pain phenotype that appears mechanistically aligned with the therapeutic target of GAE [42].

### 6.3. Patient-Reported Outcome Measures: Core Endpoints in GAE Studies

Given the symptomatic nature of GAE, patient-reported outcome measures (PROMs) constitute the cornerstone of clinical efficacy evaluation. Across published prospective cohorts and randomized trials, pain reduction and functional improvement—rather than imaging changes—represent the primary indicators of therapeutic success.

The Western Ontario and McMaster Universities Osteoarthritis Index (WOMAC) is the most widely used instrument in GAE literature. WOMAC assesses pain, stiffness, and physical function and has demonstrated consistent responsiveness to embolization, with multiple studies reporting clinically meaningful reductions exceeding established minimal clinically important difference thresholds at short- and mid-term follow-up [10,11,32,33,43]. The Knee Injury and Osteoarthritis Outcome Score (KOOS) is frequently employed in parallel or as an alternative to WOMAC. In addition to pain and function, KOOS includes subdomains addressing sport, recreational activities, and knee-related quality of life, making it particularly informative in younger or more active patients. GAE studies utilizing KOOS have reported significant improvements across pain and activities-of-daily-living subscales, with more variable responses in sport-related domains, reflecting both disease chronicity and baseline functional limitation [10,11,32,33,43].

Visual Analog Scale (VAS) pain scores are commonly used as a complementary end-point due to their simplicity and sensitivity to short-term change. The VAS typically consists of a 10-cm (100-mm) horizontal line ranging from 0 (no pain) to 10 (worst imaginable pain), often accompanied by numerical or visual descriptors to facilitate patient interpretation. Across the literature, VAS pain reduction following GAE typically ranges from 30 to 50 mm at mid-term follow-up, with a substantial proportion of patients achieving ≥ 50% pain reduction.

These outcomes appear comparable or potentially favorable compared with those reported for intra-articular injection therapies; however, differences in study design, patient populations, and follow-up duration limit direct comparisons [10,11,32,33,43,44].

### 6.4. Interpretation of Clinical Outcomes in the Context of GAE

Collectively, available data indicate that improvements in PROMs after GAE are often observed despite minimal or absent changes in structural imaging findings. This dissociation supports the concept that clinical benefit is driven by modulation of inflammatory, vascular, and nociceptive mechanisms rather than reversal of degenerative joint damage.

Accordingly, outcome interpretation in GAE should prioritize patient-centered clinical endpoints, contextualized within baseline pain severity, inflammatory imaging features, and functional impairment. Standardized use of PROMs across studies remains essential to enable meaningful comparison, meta-analysis, and refinement of patient selection criteria.

A comprehensive overview of the imaging-based and patient-reported outcome measures used to characterize OA severity and evaluate clinical response following GAE is provided in Table 2.

## 7. Pre-Procedural Clinical Evaluation and Patient Preparation

The following elements further detail the pre-procedural evaluation process within the integrated patient selection framework described above.

The pre-procedural evaluation integrates standardized clinical and functional assessment instruments, as outlined in Section 5, and plays a pivotal role in selecting appropriate candidates for GAE while systematically excluding alternative pain generators unlikely to benefit from vascular-targeted therapy [7,29].

### 7.1. Clinical History and Symptom Characterization

A detailed clinical history is essential and should document pain duration, anatomical distribution, intensity, and response to prior conservative treatments, including structured physical therapy, pharmacological management, and intra-articular injections [7,10]. Particular attention should be given to pain localization (medial, lateral, or patellofemoral compartment), mechanical versus inflammatory characteristics, activity-related exacerbation, nocturnal symptoms, and the degree of functional limitation in daily activities.

Baseline use of analgesic and anti-inflammatory medications should be systematically recorded, as post-procedural reduction in medication requirements represents a clinically meaningful secondary indicator of treatment response and has been reported in multiple GAE cohorts [30].

### 7.2. Physical Examination and Pain Mapping

Focused physical examination of the affected knee should be performed to assess joint range of motion, ligamentous stability, patellar tracking, and gait mechanics. Four-quadrant pain mapping, with areas of maximal tenderness marked using radiopaque skin markers, may be employed to facilitate correlation between clinical symptoms and angiographic vascular territories during the embolization procedure [7,8]. This clinical–angiographic correlation has been shown to assist in guiding superselective catheterization of symptomatic genicular branches and in minimizing the risk of non-target embolization, particularly cutaneous complications [11,32,37].

### 7.3. Role of Imaging in Pre-Procedural Assessment

**Figure 4:**
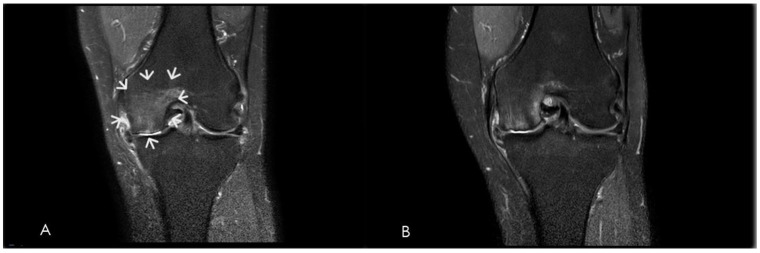
Magnetic resonance imaging (MRI) of the left knee in the same patient of Figure 3. (**A**) Pre-procedural fat-suppressed T2-weighted sequence showing synovitis and bone marrow edema (arrows) in the medial compartment. (**B**) One-month post-GAE follow-up MRI demonstrating a marked reduction in synovial hyperintensity and bone marrow edema.

Weight-bearing knee radiographs are routinely obtained to document structural osteoarthritis and establish Kellgren–Lawrence grade for descriptive staging and cohort characterization [14,15,23]. However, radiographic severity alone has limited correlation with symptom burden and should not be used as a sole determinant of eligibility for embolization [31,45]. Magnetic resonance imaging plays a pivotal role in pre-procedural evaluation by enabling exclusion of competing intra-articular and periarticular conditions that may account for knee pain and are unlikely to respond to GAE. These include osteochondral defects, osteochondritis dissecans, meniscal root tears, acute subchondral insufficiency fractures, neoplastic lesions, and significant ligamentous injuries [7,41,46]. In addition, MRI provides detailed assessment of synovitis, joint effusion, bone marrow lesions, and periarticular soft tissues, allowing comprehensive diagnostic clarification and refinement of patient selection prior to intervention (Figure 4) [41,46].

### 7.4. Assessment of Comorbidities and Procedural Risk

Relevant demographic and clinical variables should be systematically documented, including age, body mass index, cardiovascular risk factors, renal function, and the presence of peripheral arterial disease. Although elevated body mass index is a well-established risk factor for osteoarthritis development and progression, it has not been consistently associated with inferior clinical response following GAE across published cohorts [29,38]. In contrast, advanced peripheral arterial disease should be excluded, as it represents a contraindication to GAE as detailed in Section 5.2 [36,37,38].

### 7.5. Patient Counseling and Informed Consent

Informed consent should include a detailed discussion of the therapeutic rationale of GAE, procedural steps, expected benefits, potential risks, and inherent limitations of the technique [7,29]. Patients should be counseled regarding realistic expectations, including the variable temporal profile of symptom improvement, which may range from immediate post-procedural relief to gradual improvement over several weeks [10,11]. Importantly, patients should be informed that GAE does not preclude future surgical intervention and may serve as a bridge or delaying strategy rather than a definitive alternative to total knee arthroplasty, as supported by long-term follow-up data [30].

**Figure 5:**
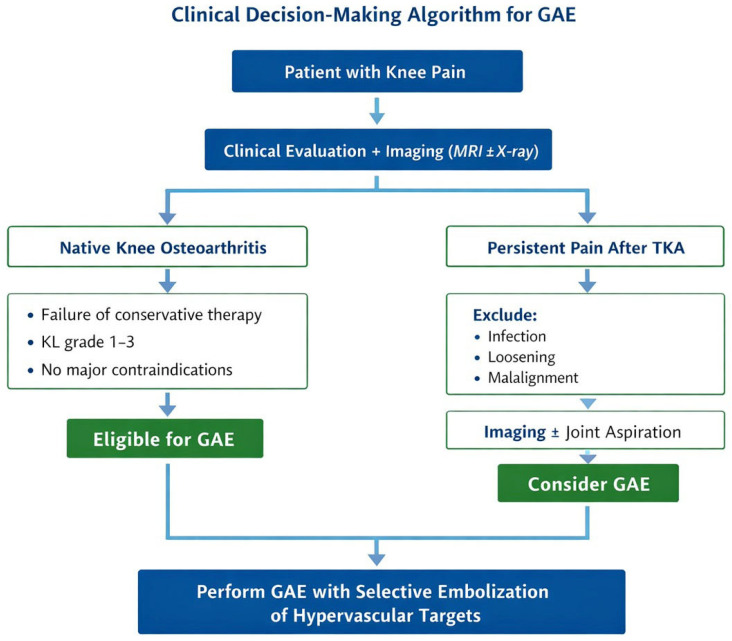
Clinical decision-making algorithm for GAE. The schematic illustrates patient selection pathways, distinguishing between native knee osteoarthritis and persistent pain after total knee arthroplasty, and highlights key clinical and imaging steps prior to treatment.

To facilitate clinical decision-making, a schematic algorithm summarizing patient selection and indication criteria for GAE is provided (Figure 5).

## 8. Anatomical Considerations in GAE

The vascular supply of the knee joint is provided by a complex periarticular arterial network, known as the genicular anastomosis, originating primarily from the distal superficial femoral artery, the popliteal artery, and the proximal anterior tibial artery. This network ensures robust collateral perfusion of periarticular soft tissues, synovium, and osseous structures and represents the anatomical substrate targeted during GAE [10,11,12,13,14,30,40,44].

**Figure 6:**
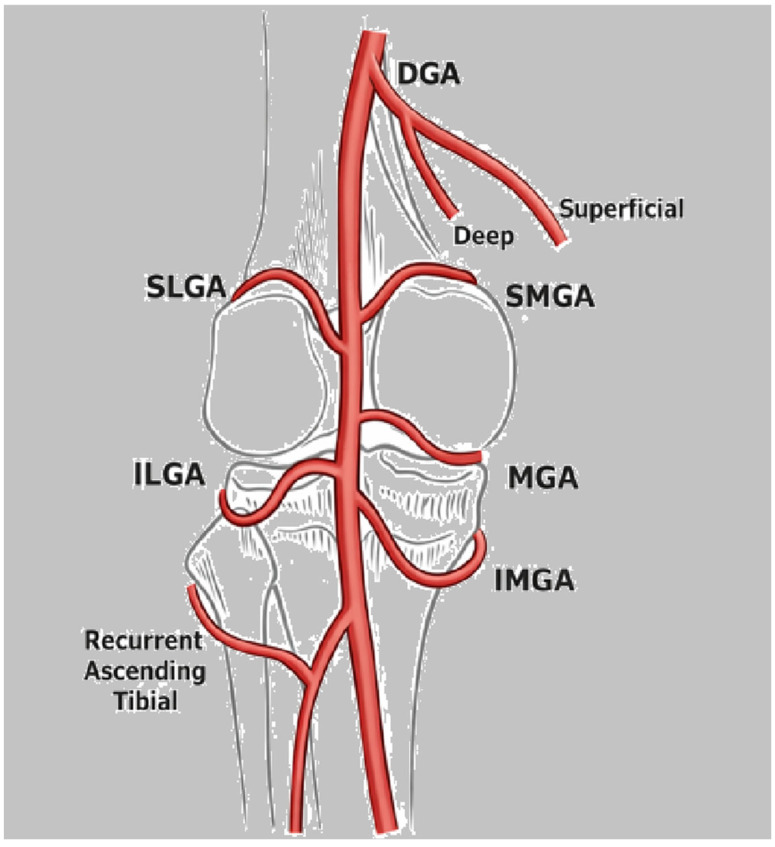
Arterial supply of the knee. Schematic representation of the genicular arterial branches relevant for selective catheterization during GAE: superior medial genicular artery (SMGA), superior lateral genicular artery (SLGA), middle genicular artery (MGA), inferior medial genicular artery (IMGA), and inferior lateral genicular artery (ILGA).

In the classic anatomical configuration, five principal genicular arteries arise from the popliteal artery: the superior medial genicular artery (SMGA), superior lateral genicular artery (SLGA), middle genicular artery (MGA), inferior medial genicular artery (IMGA), and inferior lateral genicular artery (ILGA). Additional contributions frequently originate from the descending genicular artery (DGA) and the anterior tibial recurrent artery (ATRA), particularly to the medial and anterior compartments of the knee (Figure 6).

The SMGA and SLGA course circumferentially around the distal femur, supplying the medial and lateral patellofemoral regions and adjacent periarticular tissues. The IMGA and ILGA arise near the joint line and primarily perfuse the infrapatellar fat pad, tibial plateau margins, and periarticular soft tissues [10,11,12,13,14,15,32,33,34].

Cadaveric and angiographic studies have demonstrated marked interindividual variability in the origin, course, and caliber of genicular arteries, with reported mean luminal diameters generally ranging between approximately 1.0 and 2.5 mm, depending on the specific branch and anatomical dominance. These dimensions are well suited for superselective microcatheterization and support the technical feasibility of distal embolization, while underscoring the necessity for meticulous angiographic assessment to minimize the risk of non-target embolization [47].

The MGA arises from the anterior surface of the popliteal artery and penetrates the posterior joint capsule to provide direct intra-articular vascularization. Importantly, the MGA supplies the central pivot of the knee, including the anterior and posterior cruciate ligaments, as well as adjacent synovial structures within the intercondylar notch. Given its exclusive contribution to the vascularization of critical intra-articular stabilizing structures, the MGA should be carefully identified and deliberately avoided during GAE, as it does not represent a therapeutic target and its embolization may theoretically compromise cruciate ligament integrity.

Beyond the popliteal branches, the DGA—originating from the distal superficial femoral artery near the adductor hiatus—represents a major contributor to medial compartment perfusion and is frequently implicated in medial compartment osteoarthritis. Its osteoarticular branches often represent primary embolization targets in patients with medial-sided disease [10,11,32,33,34,43,44].

The extensive collateralization among genicular branches, particularly through the patellar and periarticular plexuses, underscores the importance of detailed anatomical knowledge and superselective catheterization to maximize procedural efficacy while minimizing the risk of non-target embolization [10,23,43].

## 9. Technical Approach and Procedural Aspects

GAE is generally performed as a planned, minimally invasive procedure in an outpatient setting, under local anesthesia with optional conscious sedation. Preprocedural evaluation should include a standard angiographic workup and a careful assessment of antithrombotic therapy. Management of antiplatelet and anticoagulant agents should follow current evidence-based recommendations, with temporary modification or interruption tailored to the anticipated access-site bleeding risk and the individual patient’s thromboembolic profile [7,8,9,10].

Prior to vascular access, a focused clinical examination of the affected knee is routinely performed, and areas of maximal pain may be marked with radiopaque skin markers to facilitate angiographic–clinical correlation during the procedure [7,8]. Prophylactic measures, including intravenous antibiotics and nonsteroidal anti-inflammatory drugs, are commonly administered according to institutional protocols. Preprocedural application of ice packs over the knee has been shown to induce vasoconstriction of superficial cutaneous vessels and is associated with a lower incidence of skin-related adverse events, particularly non-target cutaneous embolization [11,32,37].

### 9.1. Vascular Access and Angiographic Mapping

**Figure 7:**
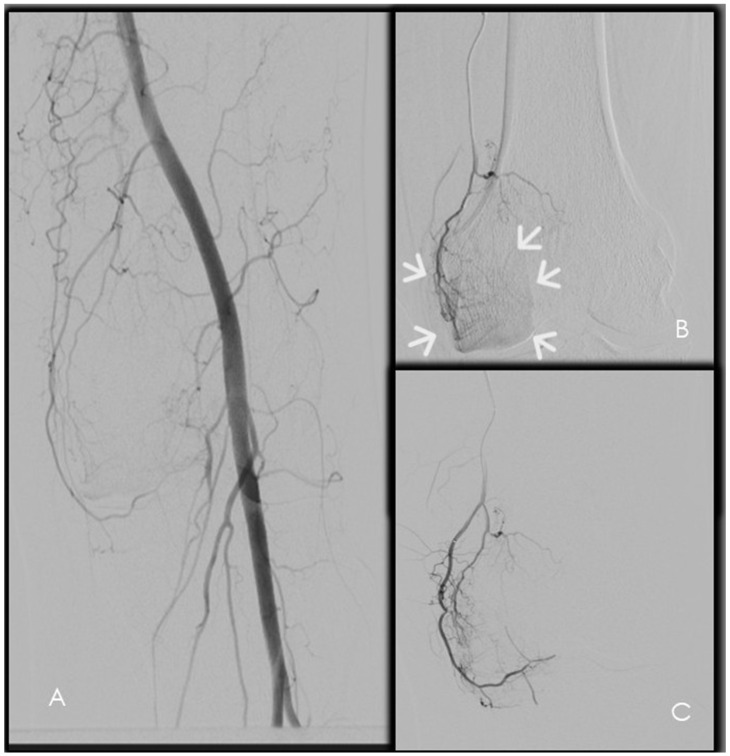
Digital subtraction angiography of the left knee. (**A**) Popliteal arteriography performed with a power injector demonstrating rich periarticular vascularization of the knee. (**B**) Selective catheterization of an osteochondral branch showing pathological hypervascular blush (arrows) in the medial compartment. (**C**) Control angiography after selective embolization with permanent particles demonstrating the characteristic “pruning effect”.

**Figure 8:**
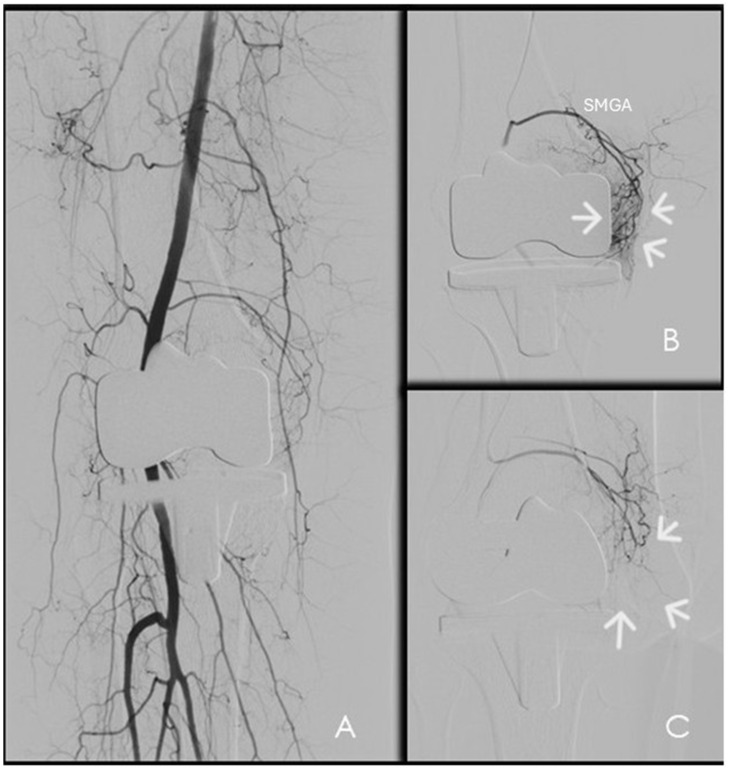
GAE in a 74-year-old female with persistent knee pain two years after total knee arthroplasty. (**A**) Digital subtraction angiography. (**B**) Selective microcatheterization of the superior medial genicular artery (SMGA) demonstrating pathological neovascularization of the medial compartment (arrows). (**C**) Final angiography after selective embolization with resorbable particles shows disappearance of hyperemia with a pruning-like effect (arrows).

Ipsilateral antegrade common femoral artery access represents the most frequently used approach, providing favorable catheter stability and ergonomic advantages for superselective catheterization of genicular branches [10,34]. Alternative access routes—including contralateral femoral, radial, or retrograde pedal access—may be selected in specific clinical scenarios based on patient anatomy, vascular status, or operator preference [7]. Initial diagnostic angiography is performed from the distal superficial femoral or popliteal artery using early and delayed digital subtraction angiography (DSA) to identify pathological periarticular hypervascularity (Figure 7). Target vessels typically demonstrate hypertrophy and abnormal synovial or periarticular blush, often corresponding to the symptomatic compartment. Subsequent selective angiography of candidate genicular branches allows detailed assessment of distal branching patterns, identification of anastomotic pathways, and refinement of the embolization strategy [7]. In selected cases—particularly in patients with prior knee arthroplasty or complex vascular anatomy—cone-beam computed tomography with intra-arterial contrast injection may provide additional value by improving visualization of small periarticular branches and clarifying vascular territories obscured by metallic artifacts [7,8,9,10] (Figure 8).

### 9.2. Embolic Agents: Permanent and Resorbable Materials

Selection of the embolic agent is a critical determinant of embolization depth, duration of vascular occlusion, modulation of inflammatory pathways, and overall safety profile. Imipenem/cilastatin sodium (IPM/CS) was the first embolic agent used in GAE and remains widely employed outside the United States. When mixed with iodinated contrast, IPM/CS forms crystalline aggregates with an effective particle size of approximately 10–70 µm, producing temporary embolization with gradual resorption and recanalization [10,11,17]. Experimental and clinical data demonstrate effective suppression of pathological hypervascularity with limited ischemic tissue injury, supporting its favorable safety profile. However, IPM/CS remains an off-label embolic agent in many jurisdictions and is not FDA-approved for this indication.

Permanent embolic microspheres, most commonly in the 75–100 µm range, are increasingly used owing to their predictable behavior, widespread availability, and regulatory approval. Commercially available materials include Embozene microspheres, Embospheres, HydroPearl, and polyvinyl alcohol (PVA) particles. These agents provide durable occlusion of pathological neovessels and have demonstrated clinical efficacy comparable to IPM/CS across multiple prospective and retrospective studies [7,11,23,34]. Some reports suggest that permanent particles may be associated with greater early pain reduction and functional improvement, potentially reflecting more sustained suppression of synovial angiogenesis in the early postprocedural phase [25]. However, mid- and long-term follow-up data indicate convergence of outcomes between permanent and temporary embolic strategies [11,33,34]. Embozene microspheres have recently obtained CE marking for musculoskeletal use, specifically for GAE in patients with knee osteoarthritis, representing the first permanent embolic agent approved in Europe for this indication.

Resorbable embolic agents represent an evolving category in GAE, designed to induce transient vascular occlusion while preserving long-term arterial patency. Biodegradable gelatin microspheres, such as Nexsphere-F, produce temporary flow reduction with resorption occurring within few hours, potentially minimizing prolonged ischemic effects (Figure 7C and Figure 8C). Nexsphere-F has received CE approval in Europe as the first embolic agent specifically indicated for arthritis embolization and has obtained FDA Breakthrough Device Designation for ongoing pivotal trials [7,10,17].

Other investigational resorbable agents—including imipenem–cilastatin–based formulations (e.g., IPZA) and biodegradable gelatin microspheres such as SakuraBead—have demonstrated encouraging early results but remain supported primarily by feasibility and early-phase studies [15,16,17,18,19,20]. Lipiodol has also been explored, mainly as an adjunctive embolic material; however, variability in embolic persistence and potential distal migration limit its role as a primary agent in GAE [21].

### 9.3. Embolization Technique and Endpoints

In keeping with the embolization philosophy described above, the goal of GAE is not complete arterial stasis but selective pruning of pathological periarticular hypervascularity while preserving antegrade flow within the parent genicular artery and maintaining collateral circulation [7,10]. Technical success is defined angiographically by resolution of abnormal synovial or periarticular blush with preservation of proximal arterial patency.

Embolic material should be injected slowly under continuous fluoroscopic monitoring, with intermittent DSA acquisitions after small-volume aliquots (typically 0.2–0.4 mL). Avoidance of forceful injections, meticulous reflux assessment, and careful evaluation of anastomotic pathways are essential to minimize the risk of non-target embolization, given the dense periarticular collateral network surrounding the knee joint.

## 10. Complications and Safety Profile

Across prospective series, randomized controlled trials, and systematic reviews, GAE has shown a reassuring safety profile, with complications typically minor, short-lived, and self-limiting, particularly when a strict superselective, distal embolization strategy is adopted. Major adverse events are uncommon in published experience to date [7,32,33,34,40,44].

### 10.1. Access-Related Events

Complications at the vascular access site mirror those of routine lower-limb endovascular procedures. Most are limited to small hematomas or minor bleeding, while pseudoaneurysm formation is rarely reported [7,40].

### 10.2. Off-Target Embolization and Skin Findings

A procedure-specific concern of GAE is inadvertent embolization of cutaneous branches, which represents the most frequently reported adverse event. Clinical manifestations may include transient erythema, livedoid changes, localized tenderness, and—less commonly—superficial ulceration or limited focal skin necrosis. Across published cohorts, the incidence of minor cutaneous complications varies widely, generally ranging between approximately 5% and 15%, depending on embolic material, embolization technique, and reporting methodology [10,11,32,34]. In the vast majority of cases, skin changes are self-limited, resolving spontaneously over several weeks without permanent sequelae, while clinically significant necrosis requiring dedicated wound care remains uncommon. Risk mitigation relies on meticulous angiographic identification of cutaneous branches, very distal microcatheter positioning, slow and controlled embolic injection with continuous reflux monitoring, and frequent angiographic reassessment. Preprocedural application of ice packs has also been associated with a reduced incidence of skin manifestations, presumably through superficial vasoconstriction [11,32,37]. In prospective cohorts using resorbable gelatin microspheres, cutaneous adverse events have been reported to be uncommon. This observation is consistent with the prospective findings reported by Taheri Amin et al., who observed no skin discoloration or other cutaneous complications in patients treated with resorbable gelatin microspheres, supporting a very low risk of skin-related adverse events with this embolic strategy [48].

### 10.3. Periprocedural and Early Post-Procedural Pain

A short-term pain flare in the hours after embolization is a recognized post-procedural pattern and is generally attributed to acute ischemic and inflammatory responses in hypervascular synovial/periarticular tissues. This phenomenon is usually self-limited and should not be interpreted as technical failure. Embolic material characteristics may influence symptom intensity: permanent particles can be associated with more pronounced early ischemic symptoms, whereas resorbable agents have been linked to lower frequency and/or shorter duration of early post-procedural pain in available reports [14,22]. When present, symptoms typically peak within 24 h and improve within 48–72 h with standard oral analgesics.

### 10.4. Neurologic Symptoms

Neurologic adverse events are uncommon and, when reported, are predominantly transient sensory symptoms (e.g., mild paresthesia or focal dysesthesia). These findings are generally interpreted as reversible ischemic/inflammatory effects involving small periarticular neural branches rather than true permanent nerve injury [7,34,40]. Aggregated experiences suggest an approximate incidence around ~1%, with persistent deficits being exceedingly rare. Meticulous angiographic mapping, avoidance of reflux, and strict superselective technique remain the key preventive measures.

### 10.5. Embolic Material and Overall Safety Considerations

Both permanent and resorbable embolic approaches appear acceptable from a safety standpoint. Permanent particles may provide more durable suppression of pathological neovascularity but can increase early ischemic-type manifestations, while resorbable materials aim for transient flow reduction with reperfusion, potentially lowering the risk of prolonged ischemic sequelae [11]. Available mid- and longer-term data show broadly comparable safety profiles, supporting case-by-case embolic selection based on anatomy, symptom pattern, and operator strategy [7,44].

While major adverse events appear broadly comparable between permanent and resorbable embolic agents, differences may exist in minor complications, particularly cutaneous events, with some evidence suggesting a lower incidence when resorbable gelatin microspheres are used.

## 11. Practical Technical Consideration and Pitfalls in GAE

From an interventional radiology perspective, procedural success in GAE is strongly influenced by technical execution and awareness of potential pitfalls that are not consistently emphasized in the literature. While most published studies focus on clinical outcomes and embolic materials, practical aspects of catheterization strategy and intraprocedural decision-making remain less systematically described.

### 11.1. Diagnostic Angiography May Underestimate Pathological Hypervascularity

Initial angiography performed from the distal superficial femoral artery using a standard 4-F diagnostic catheter (e.g., Bern or straight configuration) typically demonstrates areas of periarticular hyperemia corresponding to the embolization target. However, in some cases, pathological hypervascularity may not be clearly visible on non-selective angiography and becomes evident only after selective or superselective catheterization of individual genicular branches.

### 11.2. Stepwise Selective Catheterization Strategy

Selective catheterization of genicular arteries (SMGA, SLGA, IMGA, ILGA) is facilitated by initial engagement of the target vessel using a shaped diagnostic catheter, followed by careful advancement of a microcatheter over a microwire. A small contrast injection may help confirm vessel orientation prior to distal navigation.

### 11.3. Vasospasm Management

Vasospasm may significantly limit distal catheterization and angiographic interpretation. Intra-arterial administration of vasodilators (e.g., nitroglycerin) is recommended to improve vessel caliber and facilitate microcatheter navigation.

### 11.4. Microcatheter Navigation Without Microwire

In selected cases, advancement of the microcatheter alone—without a microwire—may be more effective, particularly in small-caliber or highly tortuous genicular branches. This is facilitated by modern microcatheter design, including braided shafts and improved pushability, which allow stable navigation while reducing the risk of microwire engagement in small collateral branches.

### 11.5. Microcatheter Tip Configuration

The use of pre-shaped or angled-tip microcatheters may facilitate selective catheterization compared with straight-tip configurations, particularly in complex or variant anatomy. Availability of a broad range of microcatheter shapes is therefore advantageous.

### 11.6. Delayed Angiographic Reassessment

After embolization, a control angiogram should be performed after a short delay. Early apparent stasis may be related to transient vasospasm rather than effective embolization. Delayed reassessment helps confirm true reduction of pathological hypervascularity and avoids premature termination of the procedure.

These technical considerations may contribute to improved procedural efficacy and reduction of non-target embolization, particularly in complex anatomical or functional scenarios (Table 3).

## 12. Clinical Evidence, Comparative Effectiveness, and Positioning of GAE

The clinical adoption of GAE is supported by an expanding body of prospective evidence, progressing from early feasibility studies to randomized controlled trials (RCTs) and long-term observational cohorts. Despite heterogeneity in study design, embolic agents, and outcome measures, available data consistently demonstrate clinically meaningful pain reduction and functional improvement in appropriately selected patients with knee OA [10,11,32,33,34,44]. An overview of the key clinical studies is summarized in Table 4.

### 12.1. Early Feasibility and Proof-of-Concept Studies

The first clinical application of GAE for OA-related knee pain was reported by Okuno et al. in 2015–2016, translating prior experience in embolization for recurrent hemarthrosis after total knee arthroplasty to a degenerative setting [10]. These pilot studies enrolled patients with mild-to-moderate OA refractory to conservative therapy and demonstrated significant reductions in pain scores and functional improvement following selective embolization of hypervascular genicular branches using imipenem/cilastatin sodium (IPM/CS) or permanent microspheres [10,11].

Although limited by small sample size, these early investigations established procedural feasibility and a favorable safety profile, with no reported cases of osteonecrosis, joint ischemia, or major ischemic complications, supporting the conceptual safety of superselective periarticular embolization [10,11].

### 12.2. Prospective Cohort Studies and Mid-Term Outcomes

Subsequent prospective cohort studies from Asia, Europe, and North America evaluated GAE in larger and more heterogeneous populations, confirming reproducible clinical benefit across different practice settings [11,12,13,14,15,16]. Consistent improvements were observed in validated patient-reported outcome measures, including VAS pain scores, WOMAC, and KOOS, often within weeks after treatment.

At mid-term follow-up (6–12 months), responder rates typically ranged between 60% and 80%, with mean VAS pain reductions of approximately 30–50 mm and WOMAC improvements exceeding minimal clinically important difference thresholds [11,32,33]. These outcomes compare favorably with those historically reported for intra-articular injection therapies, particularly in patients with imaging or clinical features suggestive of active synovial inflammation [32,33,34,43,44].

The GENESIS prospective study further strengthened mid-term evidence by demonstrating durable improvement across multiple KOOS subdomains at one year, with adverse events predominantly mild and self-limited [37]. Ongoing randomized sham-controlled trials, including the GENESIS II study, are expected to provide further high-level evidence regarding the efficacy of GAE, although results are not yet available.

### 12.3. Randomized Controlled Trials and Comparative Effectiveness

High-level evidence was reinforced by the publication of randomized controlled trials. In the first sham-controlled RCT, Bagla et al. demonstrated statistically and clinically significant reductions in pain and functional disability in patients undergoing GAE compared with controls [40]. Improvements were observed in both VAS pain scores and functional outcome measures, confirming that the therapeutic effect of GAE exceeds placebo response.

These findings position GAE favorably relative to conservative and injection-based therapies, which typically provide transient symptom relief. Subgroup analyses further suggested a dose–response relationship between the extent of embolization of pathological hypervascularity and clinical outcome, supporting the biological plausibility of the intervention [40].

### 12.4. Long-Term Outcomes, Repeat GAE, and Relationship to Surgery

Long-term follow-up data, although still limited, suggest that the clinical benefits of GAE can persist beyond one year. Two-year results from the GENESIS cohort demonstrated sustained pain reduction and functional improvement in a substantial proportion of treated patients, without evidence of delayed complications or progressive ischemic injury [30].

Importantly, patients who subsequently underwent total knee arthroplasty after insufficient or waning response to GAE did not experience increased surgical complexity or adverse operative outcomes, supporting the role of GAE as a temporizing or bridging intervention rather than a competing alternative to surgery [30]. Repeat GAE has also been reported as feasible in selected patients, with acceptable safety profiles and additional symptom improvement [15,16].

### 12.5. Summary and Positioning Within the Osteoarthritis Treatment Algorithm

Collectively, available evidence indicates that GAE provides meaningful and durable pain relief with associated functional improvement in patients with symptomatic knee OA refractory to conservative management. While heterogeneity in study design and embolic strategy limits direct comparison across trials, the consistency of benefit observed across prospective cohorts and RCTs supports GAE as an effective, mechanism-based intervention targeting vascular–inflammatory pain pathways rather than structural joint degeneration alone [10,30,32,33,34,35,43,44].

Within contemporary osteoarthritis treatment algorithms, GAE is best positioned as a minimally invasive, joint-preserving option for patients who have exhausted conservative therapies and are unsuitable for or unwilling to undergo surgical intervention, with the potential to delay—but not preclude—total knee arthroplasty.

### 12.6. Position Within Current Guidelines and Practice Recommendations

At present, GAE is not yet formally incorporated into major osteoarthritis management guidelines, including those from the American College of Rheumatology and the Osteoarthritis Research Society International. However, the growing body of prospective studies and randomized controlled trials has led to increasing recognition of GAE within the interventional radiology community.

From a clinical perspective, GAE may be considered a minimally invasive, joint-preserving option for patients with symptomatic knee osteoarthritis who have failed conservative therapies and are unsuitable for or unwilling to undergo surgical intervention. In this context, GAE can be positioned as an intermediate therapeutic option between conservative management and total knee arthroplasty, with the potential to delay surgical treatment in selected patients.

Future integration of GAE into multidisciplinary treatment algorithms and formal clinical guidelines will likely require large-scale randomized controlled trials, preferably sham-controlled, as well as standardized procedural protocols, long-term outcome data, and cost-effectiveness analyses.

### 12.7. Future Directions

Despite encouraging clinical outcomes, several areas require further investigation to fully define the role of GAE in knee osteoarthritis management. Future research should prioritize large-scale, multicenter randomized controlled trials with standardized protocols to directly compare GAE with established therapies, including intra-articular injections and genicular nerve ablation. Such studies are essential to clarify comparative effectiveness, durability of benefit, and optimal positioning of GAE within treatment algorithms [35,36,39].

Refinement of patient selection represents a critical frontier. Emerging evidence suggests that osteoarthritis phenotypes characterized by active synovitis, bone marrow lesions, and increased perfusion may respond more favorably to embolization than classifications based solely on radiographic severity. Integration of advanced imaging biomarkers—particularly MRI-based assessment of synovial inflammation, bone marrow edema, and perfusion parameters—may enable precision-driven identification of ideal candidates for GAE.

Technological innovation will continue to shape the evolution of the procedure. The development of resorbable and disease-specific embolic agents, improved microcatheter design, and advanced intraprocedural imaging techniques such as cone-beam CT and perfusion mapping may enhance procedural safety, reproducibility, and clinical outcomes. In parallel, a deeper understanding of the biological effects of transient versus permanent embolization on synovial inflammation and angiogenesis will inform embolic material selection.

Finally, long-term outcome data beyond two to three years remain limited. Future studies should address the durability of symptom relief, the role of repeat embolization, and the interaction between GAE and disease progression. Establishing evidence-based, multidisciplinary treatment pathways that integrate GAE alongside pharmacologic, rehabilitative, and surgical options will be essential for its responsible and effective adoption into routine clinical practice.

## 13. Conclusions

GAE represents a meaningful therapeutic advancement in the management of symptomatic knee osteoarthritis, particularly for patients who have failed conservative treatments and are unsuitable for or reluctant to undergo surgical intervention. The convergence of randomized controlled trials, long-term follow-up data, and growing clinical experience supports its favorable safety profile and capacity to provide sustained pain relief and functional improvement.

Optimal outcomes depend on detailed knowledge of genicular arterial anatomy, understanding of osteoarthritis pathophysiology, rigorous patient selection, comprehensive pre-procedural evaluation, and meticulous technical execution. As ongoing trials further refine patient selection and embolic strategies, GAE is increasingly positioned as an integral component of modern, multidisciplinary osteoarthritis management.

Although current evidence supports the safety and clinical efficacy of GAE, important limitations remain. In particular, long-term durability, optimal embolic strategies, and standardized patient selection criteria require further investigation through large-scale prospective studies.

## Figures and Tables

**Table 1:** Indications and Contraindications for GAE.

Category	Clinical Condition	Rationale
Indications	Symptomatic knee osteoarthritis refractory to conservative treatment	Persistent pain suggests an active inflammatory–angiogenic component potentially responsive to vascular-targeted therapy.
	Moderate-to-severe pain with radiographic OA (KL 1–4, although most evidence focuses on KL ≥ 2)	Clinical benefit appears greater in patients with higher baseline pain and active disease phenotype.
	Non-surgical candidates or patients unwilling to undergo arthroplasty	GAE offers a minimally invasive, joint-preserving option or delaying strategy.
Contraindications	Severe peripheral arterial disease	Genicular arteries may function as essential collaterals; embolization may compromise distal perfusion.
	Active knee joint infection	Vascular occlusion may impair physiological inflammatory and reparative responses
	Non-osteoarthritic or centralized pain syndromes	Pain mechanisms are unlikely to be driven by local angiogenesis, limiting therapeutic benefit.

**Table 2:** Commonly Used Imaging- and Patient-Reported Outcome Measures for the Assessment of Knee Osteoarthritis.

Scale/Score	Type	Main Domains Assessed	Score Range	Primary Clinical Use
Kellgren–Lawrence (KL)	Imaging (X-ray)	Joint space narrowing, osteophytes, subchondral sclerosis, bony deformity	Grade 0–4	Structural severity classification
WORMS(Whole-Organ MRI Score)	Imaging (MRI)	Cartilage, bone marrow lesions, synovitis, menisci, ligaments, effusion	Semi-quantitative (0–6 per feature)	Comprehensive joint-level structural assessment
MOAKS	Imaging (MRI)	Cartilage loss, BMLs, synovitis, meniscal damage	Semi-quantitative	Longitudinal OA progression assessment
WOMAC	PROM	Pain, stiffness, physical function	0–96 (or normalized 0–100; higher scores indicate worse symptoms)	Symptom severity and functional limitation
KOOS	PROM	Pain, symptoms, ADL, sport/recreation, QoL	0–100 per subscale	Expanded functional and QoL assessment
VAS Pain	PROM	Pain intensity	0–100 mm	Rapid pain quantification
OA-QoL	PROM	Impact of OA on daily life and well-being	0–100	Health-related quality of life
KOOS-Responder Criteria	Composite outcome	Pain + function improvement thresholds	Binary (responder/non-responder)	Treatment success definition

**Table 3:** Key technical tips and pitfalls in genicular artery embolization.

Domain	Key Point
Target identification	Hypervascularity may require superselective angiography
Catheterization strategy	Stepwise approach with diagnostic catheter and microcatheter
Vasospasm	Intra-arterial vasodilators may facilitate distal access
Microcatheter navigation	In selected cases, advancement without microwire may be advantageous
Microcatheter tip configuration	Angled-tip microcatheters improve navigation in complex anatomy
Procedural verification	Delayed angiographic control is recommended

**Table 4:** Key Clinical Studies Evaluating GAE for Knee OA.

Study	Year	Study Design	N (Treated/Control)	OA Severity (KL)	Embolic Agent	Follow-Up
Okuno et al. [10]	2015	Prospective pilot	14	I–III	IPM/CS ± permanent microspheres	12 mo
Okuno et al. [11]	2017	Prospective cohort	72	I–III	IPM/CS	24 mo
Lee et al. [39]	2019	Prospective cohort	48	II–IV	Permanent particles	12 mo
GENESIS (Little et al.) [30]	2021–2024	Prospective single-left trial	38	II–IV	Permanent microspheres	24 mo
Bagla et al. [40]	2023	Randomized sham-controlled trial	14/7	II–III	Permanent microspheres	1 mo
Taheri Amin et al. [48]	2025	Prospective cohort	45	II–IV	Resorbable gelatin microspheres	6 mo

## Data Availability

The original contributions presented in this study are included in the article. Further inquiries can be directed to the corresponding author.
